# Quality and performance improvement in critical care

**DOI:** 10.4103/0972-5229.42560

**Published:** 2008

**Authors:** Lakshmi P. Chelluri

**Affiliations:** **From:** Department of Critical Care Medicine, University of Pittsburgh School of Medicine, Pittsburgh PA, USA

**Keywords:** Quality, intensivist, variation, performance measures, outcomes

## Abstract

In the past decade, there is an increased focus on quality and safety in health care. Decreasing variation, increasing adherence to evidence based guidelines, monitoring processes, and measuring outcomes are critical for improving quality of care. Intensivists have broad knowledge of hospital organization, and need to be leaders in quality improvement efforts.

“…The objective of having standards is to raise them,”

*Earnest Codman*[1]

## Introduction

In the past two decades improvements in life-sustaining technologies (LST) resulted in an increase in the number of intensive care units (ICUs), and patient receiving LST in the ICUs. Care of the critically ill patients is resource-intensive, and 15-20% of hospital budgets are spent in the ICUs. The focus on quality and safety of medical care is increasing because of the high cost of health care and potential for harm.[2–5] Poor quality care is not only costly but also causes human suffering because poor quality care results in increase in morbidity and mortality. Quality Improvement (QI) initiatives in the ICU to decrease nosocomial infections and maintenance of normoglycemia have been shown to improve outcomes as well as decrease costs.[6,7] Clemmer reported that improvement in quality of care in the ICUs at a tertiary care center resulted in an estimated savings of $2.6 million per year.[8] During the past decade, in India, there are many evaluations of mortality and incidence of complications, such as nosocomial infections in the ICUs, with an increased emphasis on QI efforts and evaluation of outcomes.[9–11] Parikh *et al*, evaluated quality of care at a public hospital in Mumbai, India, and reported a higher than expected mortality which may be related to multiple deficiencies in delivery of care. In addition, the increase in travel tourism for health care to India is increasing, and there is a need to demonstrate outcomes comparable to other countries to compete effectively for this market. Public trust in health care providers could also be adversely affected if the public perceives that the care provided is not of high quality. The Medical Council of India (MCI) and the ministry of health are creating standards of care for physicians.[12] For these reasons, ICU performance need to be scrutinized closely to evaluate both the effectiveness of ICU treatments and the quality of care delivered in ICU.

The following review includes

- History of Quality Improvement
- Quality Improvement Methods and implementation
- Quality Improvement Initiatives in ICU

## History of Quality Improvement

Although there is an increase in focus on safety and quality in the past few decades, the concern about quality of health care is very old as indicated by the admonition “first do no harm”. Florence Nightingale kept records of her patients and outcomes to assess the impact of care, and suggested that knowledge of outcomes is crucial to improving care. Codman, one of the pioneers in QI in the early 20^th^ century, reported his outcomes in surgical patients and advocated pubic reporting of outcomes by both physicians and hospitals.[1] The modern QI initiatives started with recognition in other industries that unexplained variation leads to poor quality, and processes that decrease variation and continuous evaluation leads to improved quality. Shewhart and Deming were proponents of continuous evaluation of processes to improve quality and decrease defects.[13] Donabedian[14] initiated the structure, process, outcome paradigm to improve health care, and Berwick and others applied these principles to the health care and led efforts to improve quality of care in the United States of America (USA)

The report “To Err is Human” by the Institute of Medicine (IOM) in the United States in 1999, led to an increased focus on safety and quality of care. IOM suggested that care should be safe, effective, patient centered, timely, efficient, and equitable. IOM reported that that one of the primary quality problems is inappropriate use of resources, and suggested efforts to improve the use of resources by focusing on overuse, under use and misuse.[15,16] As a response to the IOM reports, many institutions initiated QI efforts to improve quality of care.

## Quality Improvement Methods and Implementation

Quality is defined by the IOM as “the degree to which health services for individuals and populations increase the likelihood of desired health outcomes and are consistent with current professional knowledge.” Although there has been more emphasis on performances of healthcare providers and quality of care recently, the focus on quality of care is not new. Codman suggested, about 100 years ago, that hospitals must collect data on their outcomes, identify strong and weak points and compare the results with other hospitals. Unexplained variation in patient care is based more on physician biases rather than patient-related factors. In the study of intensive care physicians at a university hospital, Garland *et al* reported a 43% variation in resource use and costs (≈ $1,000) between intensivists without a significant difference in mortality or length of stay.[17] Variation in care delivery makes it very difficult to monitor processes and outcomes.

Although physicians accept improving quality of care as a goal, they are sometimes skeptical of quality improvement efforts and consider participation in QI efforts as a non productive use of their time and view efforts to decrease variation as an interference with their autonomy. Physicians' behavior is influenced by suggestions from a respected colleague or role model, appropriate support for professional skill development, reinforcement by colleagues, feedback from patients, and visible results. Physicians need to agree that processes that influence clinical activity lead to measurable outcomes. Feedback and refining the process based on clinician input would get buy-in from frontline staff. Physician leaders need to be recognized as good clinicians and develop skills in communication, team building/coaching, negotiation and conflict resolution, quality improvement principles, so that they can implement QI initiatives effectively.[18–21] Physicians attempting to lead QI efforts need to be cautious on how they interact with other physicians because a wrong approach could lead to failure although the intervention is effective. The story of Ignac Sammelweis, who was a pioneer on hand hygiene but was unable to influence his colleagues, illustrates that the person who wants to initiate change needs to be able to communicate his ideas to both his superiors as well as coworkers in a non-threatening manner, and be cautious in how he conveys the message.[22] As intensive care physicians interact with many medical specialties and have a better knowledge of hospital organization because of their interaction both with the physicians as well as administrators, they are well suited to become leaders in QI initiatives.

**Figure 1:**
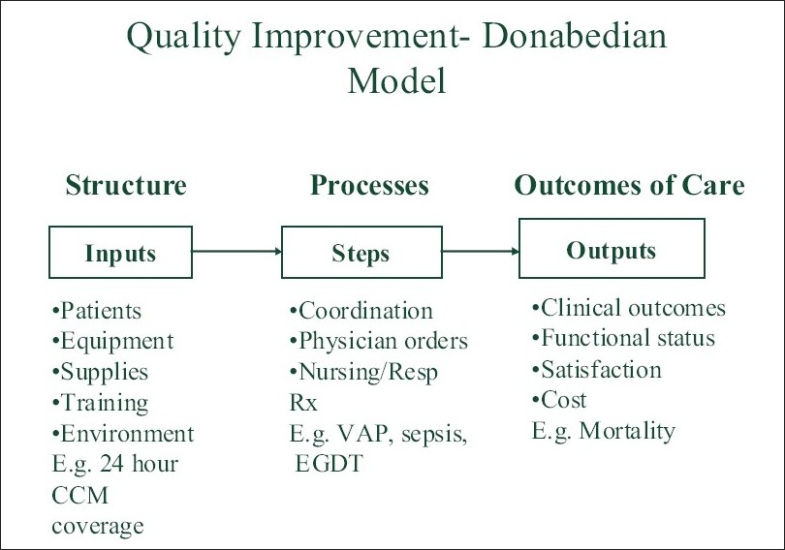
Quality Improvement- Donabedian Model

Donabedian proposed reviewing structure, process and outcome to improve quality of care.[14] The model is described in Figure 1. The Structure, in the ICU setting, refers to the type and size of the ICU, nature of staffing and availability of technology. Process issues include communication among staff, use of available technology and trainee guidelines and supervision. Outcomes include resource use, use of diagnostic and therapeutic procedures and mortality. Interventions affecting structure take longer to implement and are more expensive, so initially it is easier to target processes of care, modifying them as needed, and measuring the outcomes affected by the process. Some outcome measures such as length of stay (LOS), mortality, are easy to measure, but are affected by a number of variables and may not be easily attributed to a single intervention.

The success of QI projects depends on identifying projects which all stakeholders find useful and building a team culture. Performance measures and outcomes should be clearly defined, valid, and reliable. Documentation and data collection should be incorporated into daily work routines. Team development and process/outcome definition, followed by an iterative process of implementation, evaluation and process adjustment based on the evaluation are important steps in achieving the goals. Leadership buy-in and support is essential for implementation and success. A comprehensive plan with a description of the goals, plan for implementation, cost and benefits with business plan and timeline will be helpful in obtaining administrative support.

Successful implementation of changes in practice are facilitated by check lists, disease specific pre-printed order sets, daily order sets that include goals for care. Standardized order sets facilitate implementation of best practices in addition to improving compliance with best practices. Establishing standards of care, monitoring processes and outcomes, creation of multidisciplinary teams, data recording as part of routine care, automated retrieval of information by using information technology facilitate QI efforts. A bedside electronic record facilitates data collection and retrieval. The experience of institutions with successful implementation strategies include: leadership support, incentives for senior leaders, physician and nursing leadership in implementing the initiatives, and involvement of bedside caregivers in the design and implementation of a QI projects.

## Elements of Design and Implementation of QI Project

### Process

Identification of a clinical process that need be changed–based on benefits/risk/costs; patient care needs, informal discussions and payor priorities

### Goals

Desired outcomes.

### Personnel

Multi-disciplinary team

### Measurement

Process measures, protocol, data

### Timeline

Time needed for design, Implementation and reporting outcomes

### Reiterative process of Plan, do, Study, Act (PDSA) cycles

**Figure 2:**
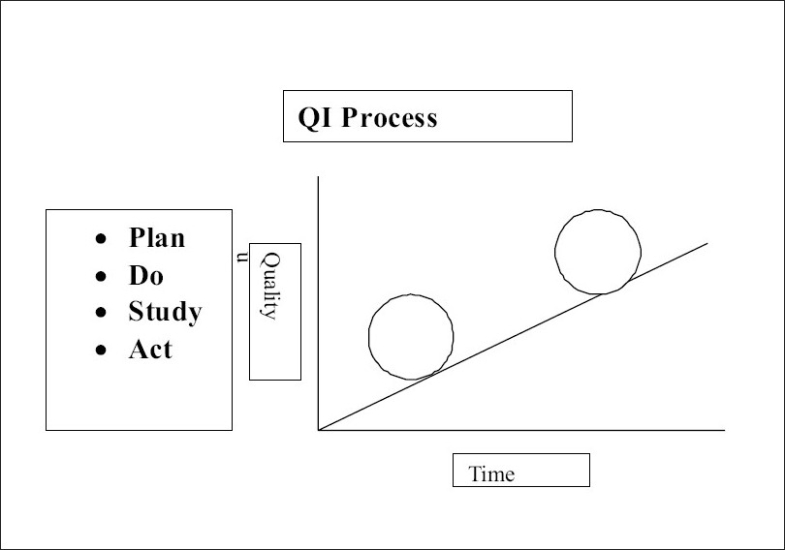
QI Process

The improvement is usually incremental and requires repeated evaluations and refinement of processes. Figure 2 illustrates the PDSA cycle

### Celebrate success

#### QI projects in the ICU

A brief summary of the initiatives, categorized according to the principles of quality listed by IOM is provided in Table 1. Table 2 contains further details of these initiatives.

**Table 1:** Attributes of quality improvement measures

Attributes	Description	Critical care examples
Safe:	Avoiding injury from care provided	Avoidable adverse events
		Medication errors
		Safety culture
Effective:	Using evidence based practices that are shown to be effective	Ventilator Associate Pneumonia (VAP), Sepsis Bundles
	Avoiding use of ineffective care, i.e. to avoid overuse, underuse, and misuse	Measures to reduce Central Line Associated Bacteremia (CLAB)
		Hand washing
		Surgical Care Improvement Project(SCIP)
		Use of Non invasive ventilation, Management of Adult Respiratory Distress Syndrome
Patient centered:	Providing care based on patient preferences, needs and values, and ensuring all clinical decisions are guided by patient's values	End of Life (EOL) Care
	Coordination and integration of care	Patient/Family satisfaction
Efficient	Avoiding waste and providing care that is shown to be effective	Blood and blood product use
		Nitric Oxide use
		Liberation from Mechanical Ventilation
Timely / Equitable	Delivering care in a timely manner and avoiding harmful delays	Patient flow; Availability of ICU beds, Avoiding use of non traditional settings to care for ICU patients
	Avoiding differences in provision care based on non medical characteristics such as gender, race, age and socio economic status	

**Table 2:** Quality improvement initiatives in critical care

Quality aims	QI project	Process measures	Outcome measures
Effective	Ventilator Associated Pneumonia (VAP)	Head of the Bed Elevation	Compliance with individual processes, incidence of ventilator associated pneumonia
		* Mouth Care, Early Appropriate diagnostic measures and antibiotic therapy	
Effective	Central Line Associated Bacteremia (CLAB)	Hand Hygiene	Compliance with individual processes and incidence of CLAB (# of infections/1000 days)-
	MRSA infections	Barrier precautions (gown, mask, hat gloves, wide barrier) Daily evaluation for the need of the catheter and early removal	
Effective	Sepsis	Early Goal Directed Therapy (EGDT), cultures, Early antibiotic therapy, Low dose steroids, Activated Protein C	Compliance with individual measures,28 day mortality
Effective/ Efficient	Sedation	Daily interruption of sedative infusions Titration of sedation to sedation/agitation goals	Compliance with individual measures, length of stay in ICU, duration of Mechanical ventilation
Efficient	Liberation from Mechanical Ventilation (MV)	Daily Spontaneous Breathing Trials (SBT)	Compliance with SBT, duration of MV, # of reintubations
Efficient	Blood transfusions	Use of transfusion guidelines: Specific transfusion trigger (E.g. Hemoglobin >7.5) Transfusion of 1 unit of RBC at a time	Compliance with trigger, Number of RBC transfusions
Effective	Glycemic control		% of patient with treatment for hyperglycemia and achieving glycemic control (Serum glucose 110-150 mg/dl)
Safe	Medical/Medication Errors	Improve reporting; Feedback to staff	# of incidents (the # may increase because currently the incidents may be under reported)
Efficient	Length Of Stay (LOS)	Patient flow:	LOS in ICU/hospital
		Appropriate discharge from ICU,	
		Early evaluation for discharge to LTAC	
Patient Centered	End Of Life Care	Appropriate communication with family on goals of therapy; modifying goals based on response to therapy IHI collaborative model	Family satisfaction, LOS
Effective			
Patient centered	Mortality		Risk adjusted mortality

**Figure 3:**
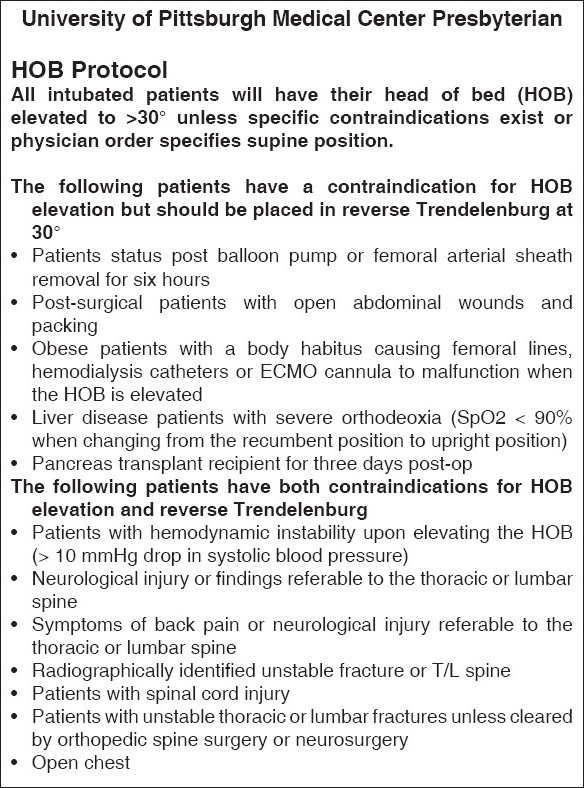
Head of bed protocol

The process should involve multidisciplinary teams consisting of intensivists, ICU nursing staff and staff of respiratory therapy department with participation from other departments such as Infection Control and Blood Bank. Consensus guidelines which include inclusion and exclusion criteria, algorithms for implementing each of the process elements, definition of outcomes and data collection need to be created, As an example of one of the projects, the algorithm for implementation of daily Spontaneous Breathing Trials (SBT) was shown in Figure 3. The experience at University of Pittsburgh Medical Center (UPMC) with SBT indicated that although the compliance with daily SBT was high, the extubation rate is not optimal. So, we are evaluating the reasons for failure to extubate and will modify the guidelines and algorithms based on the experience. It has to be noted that the success of these projects requires sustained support from the administrative and medical leadership, a physician champion, and motivated team. As patients are heterogeneous in their diseases and acuity, co-morbidities and age, any evaluation of quality needs to consider these factors. It would be helpful to collect severity of illness information so that outcomes of patients in different ICUs could be compared but it adds to the costs of obtaining data. Risk adjustment models, such as Acute Physiology And Chronic Health Evaluation (APACHE)[23] or Simplified Applied Physiology Score (SAPS),[24] adjust for these risk factors and allow comparison of different ICUs or, in some cases, evaluation of QI initiatives within a single ICU over time.

**Figure 4:**
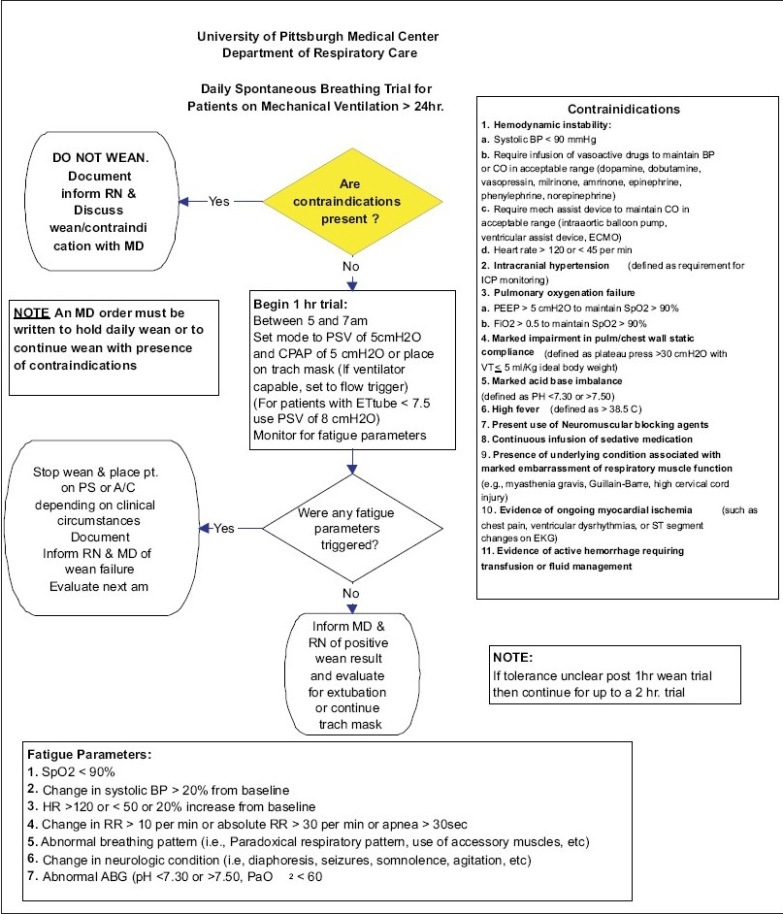
Guidelines for daily spontaneous breathing trials

**Figure 5:**
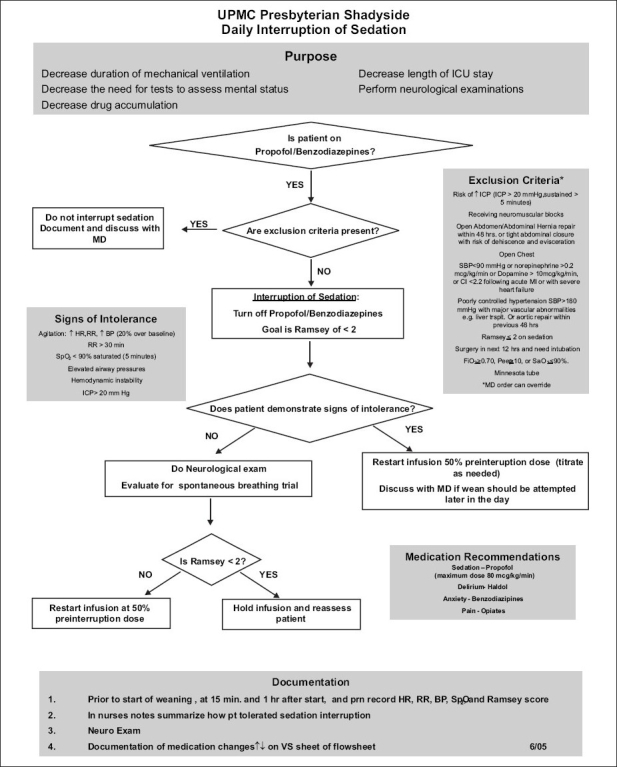
Guidelines for daily sedation interruption

**Ventilator Associated Pneumonia (VAP) bundle:** VAP increase length of stay and morbidity. Implementation of all the individual; components of the bundle has been shown to be effective in decreasing VAP.[25–26] The components are listed below and the algorithm used at UPMC is shown in Figures 3–5.

- Head of the Bed elevation (HOB) to > 30 degrees
- Daily Sedation Interruption (SI)
- Daily spontaneous Breathing Trials (SBT)
- Oral care
- Deep venous Thrombosis (DVT) prophylaxis
- Stress Ulcer Prophylaxis

Outcomes:

- Compliance with process measures (HOB, SBT, SI)
- Ventilator days, length of stay (LOS)
- Incidence of VAP - Pneumonias/1000 ventilator days
- Reintubations

**Sepsis Bundle:** Standardized management of sepsis decreases costs and improves mortality.[27] Shorr reported that mortality was 20% lower, LOS was five days shorter, and costs were ≈ $ 5,000 lower in sepsis patients treated by protocol.[28]

- Early Goal Directed Therapy (fluid resuscitation, vasopressor/ionotropic support) within six hours of identification of sepsis
- Blood cultures and other appropriate cultures prior to broad spectrum antibiotic therapy
- Imaging studies to diagnose/confirm source of infection
- Antibiotics within one hour of diagnosing sepsis
- Source control with appropriate balance of risks and benefits of chosen intervention
- Consider stress dose steroids in patients with vasopressor dependence
- Recombinant Activated Protein C in appropriate patients with in 24h of recognition of severe sepsis/shock
- Maintain hemoglobin 7-9 gm/dl if there is no active bleeding, cardiac ischemia, or hypoperfusion
- Management of adult respiratory syndrome with conservative fluid management, low tidal volumes, adequate Positive End Expiratory Pressure (PEEP) and limitation plateau pressures
- Glycemic control to maintain blood glucose < 150 mg/dl
- Reassessment and narrowing antibiotic therapy based on cultures results and limiting antibiotic use for 7-10 days.

Outcomes:

- Compliance with individual measures
- LOS in ICU and hospital
- 28 day mortality

**Central Line Associated Bacteremia (CLAB) Bundles:** Shannon *et al* reported that CLAB not only increases morbidity and but also resulted in a loss to the hospital because the reimbursement is lower than the costs. Implementation of the CLAB bundle resulted in a decrease of 825 (7.7 to 1.4 infections/1000 catheter days).[6] Pronovost *et al* reported that implementation of all elements of the bundle decrease CLAB rate from 7.7 to 1.4 infections/1000 catheter days.[29]

The components of the bundle are

- Hand Washing
- Barrier precautions (hat, mask, gown, gloves and wide cover)
- Chlorhexidine skin preparation
- Daily assessment of need for central line and early discontinuation

Outcomes:

- Compliances with individual processes
- Incidence of CLAB (# of infections/1000 days)

**Communication:** Pronovost *et al* reported that use of check list of daily goals during rounds improved communication and outcomes.[30] Discussion of daily goals during multi disciplinary rounds would help in clarifying issues and facilitate communication between staff and physicians.

Daily communication goals:

- Review labs, cultures, X-rays
- Schedule tests/procedures
- Clinical; goals for volume status, mechanical ventilation and daily
- Spontaneous breathing trials, glycemic control: location, duration
- and review for catheters/tubes
- Review of medications including antibiotic coverage
- Pain/sedation management and daily sedation interruption
- Nutrition, stress ulcer and DVT prophylaxis
- Activity
- Communication – with consultants, family

Another tool to improve communication is to standardize format of communication between staff and physicians by following the SBAR tool

- S- Situation: description of clinical situation
- B- Background: clinical history/context
- A- Assessment: a description of possible problems
- R- Recommendations: a description of possible solutions

**Rapid Response Team (RRT):** Foraida *et al* and others reported that implementation of RRT response resulted in a decrease in cardio respiratory events leading to cardiac arrest and improved survival. RRT helps to identify patients at risk and provide early resuscitation.[31] The composition of RRT is variable but usually consists of an ICU nurse, respiratory therapist and a physician skilled in airway management. The criteria for calling at RRT at University of Pittsburgh Medical Center (UPMC) are listed below

#### Respiratory

- Rate <8 or 36/ minute
- New onset difficulty breathing
- New pulse oximetry reading <85% for > 5 minutes in a patient with no prior history of chronic hypoxia
- New requirement for FiO_2_ >0.5 to obtain SaO_2_ >85%

#### Heart rate

- <40 or > 140/minute with new symptoms or any rate >160/minute

#### Blood pressure

- Systolic BP<80 or > 200 mm/hg or diastolic BP .110 mm/hg with symptoms (Neurologic change, chest pain, difficulty breathing)

#### Acute Neurologic change

- Acute loss of consciousness
- New onset lethargy or difficulty walking
- Seizure
- Sudden loss of movement or weakness of face, arm or leg

#### Other

- Chest pain unrelieved by nitroglycerin
- Unexplained agitation for > 10 minutes
- Uncontrolled bleeding, large blood loss, bleeding to airway
- Naloxone use without response

Other QI measures that could be evaluated in the ICU include:

- End-of-life care and family support
- Management of acute lung injury
- Enteral nutritional support
- Glycemic control in critically ill patients.

## Conclusion

The Institute of Medicine in the U.S. reported that there is a quality chasm in healthcare and suggested that the delivery of healthcare should be improved, so that it is safe, effective, patient-centered, timely, efficient and equitable. Both medical leadership and staff need to work together to achieve such a healthcare system. Effective implementation of existing treatments that were shown to be beneficial is more cost effective than implementing newer treatments that are marginally more effective. Intensivists, because of their broad knowledge of the hospital and interactions with multiple specialties are well suited for leading efforts to improve quality of care.
